# Dietary supplements increase the risk of excessive micronutrient intakes in Danish children

**DOI:** 10.1007/s00394-023-03153-5

**Published:** 2023-05-01

**Authors:** Camilla Christensen, Jeppe Matthiessen, Sisse Fagt, Anja Biltoft-Jensen

**Affiliations:** Research Group for Nutrition, Sustainability and Health Promotion, National Food Institute, Technical University of Denmark, Kgs. Lyngby, Denmark

**Keywords:** Nutritional supplements, Multivitamins, Minerals, Vitamins, Denmark

## Abstract

**Purpose:**

Dietary supplement use is common in Northern Europe. Many dietary supplements contain 100% of nutrient reference values (NRV) of micronutrients. This study investigates the contribution of dietary supplements to micronutrient intake, the prevalence of excess intake of micronutrients, and parental characteristics of dietary supplement use in Danish children.

**Methods:**

Data on 499 4–10-year-old children from the Danish National Survey of Diet and Physical Activity 2011–2013 were analysed using non-parametric statistics to compare micronutrient intake from the diet and dietary supplements to the reference intake (RI), and to the tolerable Upper Intake Level (UL) for users and non-users of dietary supplements. Furthermore, characteristics of the parents of users and non-users of dietary supplements were examined by logistic regression analysis.

**Results:**

Sixty-four percent of the children were dietary supplement users. Multivitamin-mineral supplements were the most frequently used type of supplement (60%). Children of never-smokers were more likely to use supplements than children of smokers. Users had significantly higher total intakes of 15 micronutrients compared to non-users. Intakes of vitamin A, zinc, and iodine from the diet alone exceeded ULs in 12–30% of the children. Use of dietary supplements gave rise to 21–73% of children in exceedance of the aforementioned three ULs as well as the UL for iron (6–45%).

**Conclusion:**

Dietary supplement use was common among 4–10-year-old Danish children and resulted in a considerable proportion of users exceeding the ULs for vitamin A, zinc, iodine, and iron. The long-term health consequences of exceeding these ULs for children are unknown.

## Introduction

Use of dietary supplements is widespread in Europe, especially in the Nordic countries, and multivitamin/mineral supplements (MVMSs) are the most commonly used type of supplement in most European countries [1–7]. Parents typically choose to administer MVMSs to their children to prevent and treat ill health and deficiencies [8–11]. This is contrary to the fact that health authorities do not recommend use of MVMSs in healthy children. In Denmark, health authorities recommend only vitamin D supplementation year-round for children under 4 years, iron for children born prematurely, and calcium for children allergic to dairy [12].

In Europe, the content of vitamins and minerals in dietary supplements must be declared as a percentage of the nutrient reference value (NRV). NRVs are common across all European countries and represent the average requirement for vitamins and minerals for children and adults. However, the average requirement encompasses large variation, since requirements differ based on gender, age group, and other factors [1]. Supplement manufacturers use the European NRVs as a reference, and the majority of multivitamin/mineral supplements registered in Denmark contain 100% or more of the NRVs for most micronutrients [13, 14]. Thus, by taking a daily multivitamin/mineral product on top of an adequate diet, there may be a risk of Danes exceeding their requirements for vitamins and minerals. Children are especially at risk of consuming micronutrients at levels above the upper intake levels (ULs), since the margins between recommended levels of daily intake and ULs are smaller for children than for adults. However, little is known about how the common European NRVs fit with dietary habits in individual European countries such as Denmark, and how dietary supplements affect the total micronutrient intake of children specifically. Whilst data on dietary supplement use by Danish adults and adolescents has been published [3, 13, 15], there is a paucity of data on dietary supplement use by Danish children. The aim of the present study was to examine the use of dietary supplements in a nationwide sample of Danish children aged 4–10 years and assess the risk of excessive micronutrient intakes, as well as to determine the predictors of dietary supplement use.

## Methods

### Participants

A random sample was drawn from the Danish Civil Registration System [16] in order to obtain a representative sample of the Danish population. The present study comprises 499 children aged 4–10 years. The response rate was 54% and 67% for the entire survey population (age 4–75 years) and for 4–14-year-olds, respectively [17]. The response rate was not calculated for the age range 4–10 years. The study population was divided into two age groups: 4–6-year-olds and 7–10-year-olds. As children aged ≥ 11 years are recommended the same dose of vitamins as adults, this study only includes children aged 4–10 years. Verbal consent was obtained from the parents of the children.

### Data collection

Data are derived from the Danish National Survey of Diet and Physical Activity (DANSDA) 2011–13, which is a nationwide cross-sectional survey. The methods of the survey are described in more details elsewhere [17]. Briefly, information was collected on the parents’ socio-economic status and health beliefs and on the child’s lifestyle, and anthropometric measurements were taken on the children during a face-to-face interview. Data were collected from April 2011 to August 2013. Dietary data were collected by means of a validated 7-day pre-coded dietary record using household measures and series of photographs to estimate portion sizes [18, 19]. Intakes of nutrients from the diet (including iodine from mandatory fortification of salt) were calculated using the software system General Intake Estimation (GIES) version 1.000.i6 and the Danish Food Composition Database version 7.0 (www.foodcomp.dk). The Danish Food Composition database is hosted at the National Food Institute, Technical University of Denmark and updated regularly. Samples of food and drinks are collected throughout the year to account for seasonal variation in the content of nutrients. The products analysed are selected based on retail GfK ConsumerScan’s household purchasing data. Average daily intakes of foods and nutrients were estimated for each participant. Participants were included in the data analysis if they had recorded their diet for at least four days.

As part of the face-to-face interview, information on use of dietary supplements in the past year was collected, including use of MVMS, vitamin C, fish oil/cod liver oil, iron, calcium with or without vitamin D, vitamin D and ‘other types’ of dietary supplements. The question about ‘other types’ was open-ended. MVMS were defined as products containing three or more vitamins with or without minerals and other active ingredients (e.g. fish oil, probiotics or herbs) [20–22]. Exceptions were protein supplements containing vitamins and/or minerals and products containing B vitamins only. In approximately 90% of interviews, the mother of the participating child was interviewed. Brand names as well as consumption frequency were recorded for the different supplements used. However, for multivitamin/mineral products no brand names were collected since the products in this category were similar. Information was collected on the daily and month-to-month frequencies of supplement intake, as well as on the dose. Nutrient composition of the supplements reported in the present study were obtained from the Danish Veterinary and Food Administration’s database of registered dietary supplements [23]. Product information not included in the database was obtained from direct communication with supplement manufacturers, supplement labels or Internet websites. For products reported by participants with insufficient detail to match a known product, generic nutrient profiles were assigned, e.g. a generic vitamin D supplement for vitamin D products reported without the brand name. These generic nutrient profiles were created using a weighted average according to frequency of use of each product reported for every supplement type. Additionally, a generic MVMS were created based on GfK ConsumerScan’s household purchasing data for MVMSs. The GfK ConsumerScan household panel was comprised of about 3,000 representative Danish households, and data reflect real purchasing behaviour of individual households over extended periods [3]. GfK data from 2011 to 2013 was used.

Participants were classified as supplement users if they reported taking at least one dietary supplement within the last year, irrespective of the frequency of use and whether the product contained vitamins, minerals, or both. For assessment of individual micronutrients, participants were classified as users if they consumed a supplement containing the given micronutrient. Those who did not consume the particular nutrient in supplemental form were classified as non-users of that nutrient, even if they consumed a supplement containing other nutrients. In this paper,’dietary intake’ refers to micronutrients obtained from foods and drinks and ‘total intake’ refers to the sum of micronutrients from food, drinks, and dietary supplements.

Tolerable Upper Intake Level (UL) values established by the Scientific Committee on Food (SCF) and EFSA (European Food Safety Authority) were used to assess the proportion of children at potential risk of excessive nutrient intakes [24]. Where no UL values were listed by SCF, guidance levels (GLs) or temporary guidance levels (TGLs) were applied as suggested by a British [25] and a Danish expert group, respectively [26]. The proportion of participants whose intake of individual micronutrients exceeded the UL and whose intake exceeded the UL by 150% were calculated.

### Statistical analyses

All statistical analyses were performed in SPSS (version 25, IBM SPSS Statistics). Continuous variables were not normally distributed and therefore groups were compared using the Mann–Whitney U test. The relationship between categorical variables and supplement use was explored by a Chi-squared test. When the expected number was less than five in a group, the Fisher’s exact 2-sided P-value is reported. When the independent variable included two categories, the continuity correction P-value is reported. In cases with more than two categories, the Pearson P-value is reported. Proportions are expressed as percentages and the corresponding 95% confidence intervals (CIs) were calculated using the Clopper-Pearson method.

Associations between dietary supplement use (user/non-user) and education (highest in household), smoking status, self-perceived dietary habits, intention to eat healthily, self-perceived health, age, sex, BMI, physical activity, fruit and vegetable intake and fish intake were examined by binary logistic regression analysis. A stepwise backward logistic regression approach was used, and all explanatory variables were included as categorical variables. Only variables significantly associated with supplement use were included in the final model. The results are presented as odds ratios (ORs) and 95% CIs. Collinearity diagnostics showed no indications of dependence between the factors included. A P-value less than 0.05 was considered significant.

Data on education, smoking status, self-perceived dietary habits and health, intention to eat healthily pertained to the parent of the participating child. The parent was asked if his/her dietary habits were sufficiently healthy and could answer: ‘not at all’, ‘partly’, ‘to some extent’ or ‘very much’. Intention to eat healthily was measured as ‘never’, ‘sometimes, ‘often’, or ‘very often’. Some categories of BMI, education, smoking status, and self-perceived health were merged prior to statistical analyses due to low number of participants in some categories. The highest educational attainment in the household was divided into two categories: ‘basic school/vocational education’ and ‘higher education’. As for parental smoking status, the ‘smoker’ category included those who smoked every day and those who smoked occasionally. The two other categories were ‘never-smokers’ and ‘former smokers’. After merging the categories ‘not so good’ and ‘poor’ self-perceived health answers were divided into four categories: ‘excellent’, ‘very good’, ‘good’ and ‘not so good or poor’. Continuous variables for physical activity, BMI, fruit and vegetable intake and fish intake’ were converted into categorical variables. Physical activity (pedometer-determined steps per day) had four categories: < 5000, 5000–9999, 10.000–14.999 and ≥ 15,000 steps/day. Fruit and vegetable intake was divided into three categories: < 200, 200–399.9, and ≥ 400 g/10 MJ/day. Fish intake was split into four categories: < 50, 50–199.9, 200–349.9 and ≥ 350 g/10 MJ/week. BMI had two categories ‘underweight/normal weight’ and ‘overweight/obesity’. The International Obesity Task Force age-specific and gender-specific BMI cut-off points for normal weight, overweight, and obesity were used [27, 28].

To assess dietary quality, the diet of each participant was given a diet quality score (Danish Diet Quality Index). This was based on how well their diet complied with five of the official Danish dietary guidelines for fruit and vegetables, fish, whole grain, saturated fatty acids, and added sugars. The calculation method of this score is published in detail elsewhere [29].

The ratio between energy intake and basic metabolic rate was used to classify the participants as under-reporters (EI/BMR < 1.121), plausible reporters (2.892 > EI/BMR ≥ 1.121), and over-reporters (EI/BMR ≥ 2.892) of energy intake. BMR was estimated using the gender and age-specific Schofield equations [30].

## Results

### Characteristics of the study population

Sixty-four percent of the 499 children were users of dietary supplements (Table 1). There was no difference in the proportion of supplement users between boys and girls, the two age groups, educational attainment, self-rated health, dietary quality score or median BMI of the child or parent. There was a significantly smaller proportion of smokers and a higher proportion of never-smokers among the supplement users (*P* = 0.003). The results of the logistic regression analysis confirmed this, as smoking status was the only significant independent variable associated with dietary supplement use (Table 2). Parental education (highest in household), intention to eat healthily (parent), self-perceived dietary habits (parent), sex, BMI, physical activity (pedometer-determined steps per day), fruit and vegetable intake and fish intake showed no association with use of dietary supplements (data not shown).

**Table 1:** Descriptive characteristics of the study population (median or % and 95% CI)

	n	Non-user	User	P-value
Total n (%, 95% CI)	499	36.3 (32.0–40.7)	63.7 (59.3–68.0)	
Sex				
Girls (%, 95% CI)	248	51.4 (43.9–58.9)	48.7 (43.1–54.4)	0.636
Boys (%, 95% CI)	251	48.6 (41.1–56.1)	51.3 (45.6–56.9)	
Age groups				
4–6 years (%, 95% CI)	203	37.0 (30.0–44.5)	42.8 (37.3–48.4)	0.245
7–10 year (%, 95% CI)	296	63.0 (55.5–70.0)	57.2 (51.6–62.7)	
BMI (median, 95% CI)	465	16.6 (14.0–20.7)	16.3 (13.8–19.8)	0.147
BMI (%, 95% CI)				
Underweight/normal weight	389	80.9 (74.0–86.6)	85.1 (80.6–89.0)	0.290
Overweight/obese	76	19.1 (13.4–26.0)	14.9 (11.0–19.4)	
Parent’s BMI (median, 95% CI)	489	24.1 (19.8–31.2)	24.1 (19.5–34.6)	0.858
Parent’s BMI (%, 95% CI)				
Underweight	10	2.3 (0.6–5.7)	1.9 (0.7–4.1)	0.357
Normal weight	288	58.5 (50.9–65.9)	59.1 (53.4–64.6)	
Overweight	120	27.8 (21.4–35.1)	22.7 (18.2–27.7)	
Obese	71	11.4 (7.1–17.0)	16.3 (12.4–20.9)	
Highest education in household (%, 95% CI)	180	37.0 (30.0–44.5)	35.9 (30.6–41.4)	0.874
Basic school or vocational education (≤ 13 years) Higher education (≥ 13 years)	316	63.0 (55.5–70.0)	64.1 (58.6–69.4)	
Self-perceived health (%, 95% CI)				
Excellent	143	26.0 (19.7–33.0)	30.3 (25.3–35.7)	0.435
Very good	215	47.0 (39.5–54.5)	41.0 (35.5–46.6)	
Good	112	20.4 (14.8–27.1)	23.7 (19.1–28.7)	
Not so good or poor	28	6.6 (3.5–11.3)	5.0 (2.9–8.1)	
Diet quality index (median, 95% CI)	499	3.34 (1.85–4.68)	3.31 (1.89–4.43)	0.645
Diet quality index quartiles (%, 95% CI)				
< 25% (Scores < 2.86)	125	27.6 (21.3–34.7)	23.6 (19.0–28.6)	
25–75% (Scores 2.86–3.85)	250	44.2 (36.8–51.8)	53.5 (47.8–59.0)	0.136
> 75% (Scores > 3.85)	124	28.2 (21.8–35.3)	23.0 (18.4–28.0)	
Smoker (parent) (%, 95% CI)				
Yes, smoker	78	22.1 (18.4–33.1)	12.0 (8.6–17.3)*	0.003
No, former smoker	128	28.2 (21.8–38.0)	24.3 (21.8–34.1)	
No, never smoked	292	49.7 (37.1–54.7)	63.7 (53.4–66.6)*	

Groups were compared with a Mann–Whitney U-test or χ^2^ test

*P < 0.05 (Non-user vs user)

**Table 2:** Variables significantly associated with the use of dietary supplements in Danish children aged 4–10 years

	Total *n*	User *n* (%)	OR	95% CI	*P*-value
Smoker (parent)					
No, never smoked	248	174 (70)	1.00		0.001
No, former smoker	108	65 (60)	0.50	0.26–0.96	0.036
Yes	58	25 (43)	0.32	0.18–0.58	< 0.0001

OR: odds ratio, CI: confidence interval. Stepwise backward logistic regression analysis (*P* < 0.05). N = 414. Due to missing data for one or more of the variables, 85 individuals were excluded from the analysis. Variables included in the regression analysis: age, sex, BMI, physical activity, fruit and vegetable intake, fish intake, intention to eat healthily, smoking habits, education, self-perceived health and dietary habits

### Types and number of dietary supplements

MVMSs were by far the most common type of supplement with 60% of users reporting taking a MVMS, and fish oil was the second most popular type of supplement (Table 3). Seventeen percent reported taking other types of supplements, including single vitamin and mineral supplements, fish oil, yeast-based supplements, dietary fibre supplements, probiotics, protein supplements and herbal/botanical products. Fifty percent consumed one dietary supplement and 12% consumed two dietary supplements (Table 4). Only a small proportion of the children consumed three or four dietary supplements (≤ 2%). There were no significant differences in use of the different types of supplements or the number of dietary supplements taken between the two age groups or between boys and girls.

**Table 3:** Types of supplements used among 4–10-year-old children^a^

	n	Multivitamin/mineral	Vitamin C	Calcium (± Vitamin D)	Vitamin D	Fish oil	Other dietary supplements^b^
Total	499	60.3 (55.9–64.6)	1.2 (0.4–2.6)	2.8 (1.5–4.7)	2.0 (1.0–3.7)	8.6 (6.3–11.4)	5.2 (3.4–7.5)
Sex							
Boys	251	62.2 (55.8–68.2)	0.8 (0.1–2.8)	3.2 (1.4–6-2)	2.4 (0.9–5.1)	10.0 (6.5–14.4)	4.4 (2.2–7.7)
Girls	248	58.5 (52.1–64.7)	1.6 (0.4–4.1)	2.4 (0.9–5.2)	1.6 (0.4–4.1)	7.3 (4.4–11.2)	6.0 (3.4–9.8)
Age							
4–6 years	203	64.0 (57.0–70.6)	1.0 (0.1–3.5)	3.9 (1.7–7.6)	1.5 (0.3–4.3)	8.9 (5.3–13.7)	5.4 (2.7–9.5)
7–10 years	296	57.8 (51.9–63.5)	1.4 (0.4–3.4)	2.0 (0.7–4.4)	2.4 (1.0–4.8)	8.4 (5.5–12.2)	5.1 (2.9–8.2)

^a^None of the 499 participants reported use of iron supplement and thus iron is omitted from the table

^b^Supplements not included in the Multi-Vitamin-Mineral, vitamin C, vitamin D, calcium or fish oil categories

**Table 4:** Number of supplements used by children aged 4–10 years^a^

Number of supplements	n	% (95% CI)			
		1	2	3	4
Total	499	50.1 (45.6–54.6)	11.6 (8.9–14.8)	1.0 (0.3–2.3)	0.8 (0.2–2.0)
Sex					
Boys	251	50.6 (44.2–56.9)	11.6 (7.9–16.2)	1.2 (0.2–3.5)	1.2 (0.2–3.5)
Girls	248	49.6 (43.2–56.0)	11.7 (8.0–16.4)	0.8 (0.1–2.3)	0.4 (0–2.2)
Age					
4–6 years	203	53.7 (46.6–60.7)	10.3 (6.5–15.4)	1.5 (0.3–4.3)	1.5 (0.3–4.3)
7–10 years	296	47.6 (41.8–53.5)	12.5 (9.0–16.8)	0.7 (0.1–2.4)	0.3 (0–1.9)

^a^Including non-vitamin-mineral supplements (e.g., fish oil, herbal and yeast based products)

### Micronutrient intakes and proportions of study population in exceedance of ULs

There were no significant differences in dietary intakes of 17 selected micronutrients among users and non-users (Table 5). However, dietary supplement users had significantly higher median total intakes of 15 of these 17 micronutrients compared with non-users. Total calcium and total magnesium intakes were not significantly different between dietary supplement users and non-users, although for calcium it was borderline significant (*P* = 0.057, n = 499).

**Table 5:** Energy, macronutrient, dietary and total vitamin and mineral intakes (mean, 5th percentile, median and 95th percentile) in dietary supplement users and non-users among children aged 4–10 years

		Non-users					Users									
		Dietary intake^a^					Dietary intake					Total intake^b^				RI (6–9 years)[60]
		n	Mean (SD)	5%	50%^c^	95%	n	Mean (SD)	5%	50%	95%	Mean (SD)	5%	50%^d^	95%	
Energy	MJ/day	181	8.3 (1.4)	5.6	8.0	11.4	318	8.2 (1.0)	5.3	8.0	11.5	–	–	–	–	–
Carbohydrate	E%	181	50.9 (0.3)	43.6	51.3	60.0	318	50.7 (0.3)	43.3	50.7	57.8	–	–	–	–	–
Protein	E%	181	14.6 (0.2)	11.4	14.7	18.4	318	14.3 (0.1)	11.1	14.1	18.2	–	–	–	–	–
Fat	E%	181	34.5 (0.3)	26.7	33.8	43.2	318	35.0 (0.2)	27.6	35.2	42.2	–	–	–	–	–
Saturated fat	E%	181	13.8 (0.2)	10.1	13.8	18.1	318	14.0 (0.1)	10.6	14.0	17.8	–	–	–	–	–
Dietary fibre	E%	181	19.8 (0.4)	11.0	19.7	28.1	318	19.8 (0.3)	11.6	19.1	30.0	–	–	–	–	–
Micronutrient																
Vitamin A	RE	195	1182 (45)	403	1075	2497	304	1220 (40)	395	1057	2645	1497 (41)	651	1368***	2853	400
Retinol	µg	195	811 (39)	224	659	1809	304	858 (36)	232	673	2115	1112 (40)	377	912***	2368	N/A
Vitamin D	µg	189	2.8 (0.1)	1.0	2.2	6.9	310	2.8 (0.2)	0.97	1.94	5.65	9.6 (0.3)	2.5	9.4***	20.0	10
Vitamin E	α-TE	193	7.3 (0.2)	4.1	7.0	12.0	306	7.6 (0.2)	4.1	7.0	13.1	12.2 (0.2)	6.6	12.2***	19.2	6
Thiamin	mg	195	1.21 (0.02)	0.73	1.16	1.81	304	1.19 (0.02)	0.71	1.14	1.77	1.7 (0.0)	1.0	1.7***	2.4	0.9
Riboflavin	mg	195	1.64 (0.04)	0.83	1.59	2.81	304	1.59 (0.03)	0.91	1.54	2.48	2.2 (0.0)	1.3	2.2***	3.1	1.1
Niacin	NE	195	23.0 (0.5)	12.7	22.4	35.5	304	21.9 (0.3)	12.9	21.4	33.2	27.6 (0.4)	18.3	27.1***	39.5	12
Vitamin B6	mg	195	1.28 (0.03)	0.75	1.25	1.91	304	1.21 (0.02)	0.76	1.17	1.82	1.9 (0.0)	1.1	1.9***	2.7	1.0
Folic acid/folate	mg	198	285 (6)	165	270	431	301	279 (5)	162	264	436	338 (5)	217	325***	505	130
Vitamin B12	µg	197	5.4 (0.2)	2.2	5.0	10.5	302	5.3 (0.1)	2.3	5.0	9.6	6.2 (0.1)	3.1	5.9***	10.3	1.3
Vitamin C	mg	195	106 (4)	44	92	209	304	102 (3)	46	91	187	138 (4)	64	126***	231	40
Calcium	mg	194	1035 (25)	523	965	1688	305	1010 (17)	598	965	1601	1082 (18)	638	1285	1639	700
Magnesium	mg	198	294 (5)	178	288	422	301	289 (5)	184	275	441	304 (5)	198	288	450	200
Iron	mg	198	9.1 (0.2)	5.7	9.0	13.1	301	9.0 (0.1)	5.8	8.7	13.8	13.8 (0.2)	8.6	13.8***	19.7	9
Zinc	mg	198	9.7 (0.2)	5.8	9.3	15.1	301	9.4 (0.1)	6.0	9.1	14.1	12.3 (0.2)	8.2	12.1***	17.0	7
Iodine	µg	198	234 (9)	97	204	449	301	216 (6)	109	191	437	258 (6)	130	237***	490	150
Selenium	µg	198	41.0 (0.9)	23.4	38.9	64.0	301	39.0 (0.7)	23.4	37.3	59.3	51.4 (0.8)	32.4	50.0***	74.0	30

*p < 0.05; ** p < 0.01; *** p < 0.001

^a^Micronutrients from foods and drinks only

^b^Micronutrients from foods, drinks, and supplements

^c^Differences in median dietary micronutrient intakes between non-users and users of the specific micronutrient were tested with a Mann–Whitney U test. Only significant differences are indicated

^d^*P*-values for the difference in median dietary micronutrient intake (non-users) and total micronutrient intake (users)

In both age groups, dietary intakes of vitamin A (retinol), zinc and iodine exceeded ULs in 11.5–30.1% of the children (Tables 6 and 7). When considering the total retinol intake in 4–6-year-old users, 43.1% exceeded the UL for retinol and 16.9% exceeded the UL by 150%. The latter proportion was significantly different from the proportion of non-users exceeding the UL by 150% (4.1%, *P* = 0.015). Furthermore, supplement use resulted in 45.4% of children exceeding the TGL for iron (total intake in users vs non-users, *P* < 0.001). For 73% of 4–6-year-old users, total zinc intake exceeded the UL and for 10.8% it exceeded the UL by ≥ 150%, which is significantly different from non-users (0%, *P* = 0.009).

**Table 6:** Percentages of users and non-users of specific micronutrients among children aged 4–6 years (n = 203) in exceedance of the upper intake level (UL) through the diet alone and when also considering supplement use (% (95% CI)) and the mean (SD) and median (95% CI) intakes of micronutrients

Micronutrient		UL	Non-users		Users				Non-users		Users				
			Dietary intake		Dietary intake		Total intake		Dietary intake		Dietary intake		Total intake		
			> UL	> 150%	> UL	> 150%	> UL	> 150%	Mean (SD)	Median (95%CI)	Mean (SD)	Median (95% CI)	Mean (SD)		Median (95% CI)
Retinol	µg	1100	30.1 (19.9–42.0)	4.1 (0.9–11.5)	28.5 (20.9–37.0)	8.5 (4.3–14.6)	43.1 (34.4–52.0)	16.9 (10.9–24.5)*	832 (59)	779 (182–1714)	854 (48)	688 (231–1942)	1127 (50)		983 (377–2287)***
Vitamin D	µg	25^a^	0 (0.0–4.9)	0 (0.0–4.9)	0 (0.0–2.8)	0 (0.0–2.8)	0.8 (0.0–4.2)	0 (0.0–2.8)	2.4 (0.2)	2.0 (0.8–6.0)	2.6 (0.2)	1.9 (0.9–6.8)	9.9 (0.4)		10.8 (2.5–19.7)***
Vitamin E^b^	α-TE^c^	120	–	–	–	–	0 (0.0–2.8)	0 (0.0–2.8)	6.7 (0.2)	6.6 (3.8–10.9)	7.2 (0.3)	6.6 (3.9–13.1)	12.3 (0.4)		12.4 (6.9–19.1)***
Thiamin	mg TGL^d^	20	0 (0.0–4.9)	0 (0.0–4.9)	0 (0.0–2.8)	0 (0.0–2.8)	0 (0.0–2.8)	0 (0.0–2.8)	1.1 (0.0)	1.1 (0.7–1.5)	1.1 (0.0)	1.1 (0.7–1.6)	1.7 (0.0)		1.7 (1.0–2.4)***
Riboflavin	mg GL^e^	16	0 (0.0–4.9)	0 (0.0–4.9)	0 (0.0–2.8)	0 (0.0–2.8)	0 (0.0–2.8)	0 (0.0–2.8)	1.5 (0.1)	1.5 (0.8–2.4)	1.5 (0.0)	1.5 (0.9–2.3)	2.1 (0.0)		2.1 (1.3–3.1)***
Niacin	NE^f^	220	0 (0.0–4.9)	0 (0.0–4.9)	0 (0.0–2.8)	0 (0.0–2.8)	0 (0.0–2.8)	0 (0.0–2.8)	20.2 (0.6)	19.4 (12.5–29.3)	20.0 (0.5)	18.9 (12.0–31.5)	26.2 (0.5)		25.6 (12.5–29.4)***
Vitamin B6	mg	7	0 (0.0–4.9)	0 (0.0–4.9)	0 (0.0–2.8)	0 (0.0–2.8)	0 (0.0–2.8)	0 (0.0–2.8)	1.2 (0.0)	1.1 (0.7–1.7)	1.1 (0.0)	1.1 (0.7–1.6)	1.9 (0.0)		1.9 (1.1–2.6)***
Folic acid^b^	mg	300	–	–	–	–	0 (0.0–2.8)	0 (0.0–2.8)	266 (9)	247 (163–411)	267 (6)	259 (154–394)	331 (9)		327 (234–453)***
Vitamin B12	µg GL^e^	730	0 (0.0–4.9)	0 (0.0–4.9)	0 (0.0–2.8)	0 (0.0–2.8)	0 (0.0–2.8)	0 (0.0–2.8)	5.1 (0.2)	5.0 (2.3–8.9)	5.0 (0.2)	4.8 (2.3–8.4)	5.9 (0.2)		5.6 (3.4–9.5)**
Vitamin C	mg TGL^d^	370	0 (0.0–4.9)	0 (0.0–4.9)	0 (0.0–2.8)	0 (0.0–2.8)	0.8 (0.0–4.2)	0.8 (0.0–4.2)	95 (6)	82 (41–205)	102 (4)	94 (47–180)	139 (5)		131 (70–220)***
Calcium	mg	2500	0 (0.0–4.9)	0 (0.0–4.9)	0 (0.0–2.8)	0 (0.0–2.8)	0 (0.0–2.8)	0 (0.0–2.8)	945 (32)	912 (489–1412)	927 (22)	897 (558–1392)	1007 (24)		962 (617–1486)
Magnesium ^b^	mg	250	–	–	–	–	0 (0.0–2.8)	0 (0.0–2.8)	268 (6)	266 (171–373)	270 (6)	261 (174–396)	287 (6)		277 (194–415)
Iron	mg TGL^d^	14	0 (0.0–4.9)	0 (0.0–4.9)	0.8 (0.0–4.2)	0 (0.0–2.8)	45.4** (36.6–54.3)	2.3 (0.5–6.6)	8.1 (0.2)	8.2 (5.4–11.5)	8.4 (0.2)	8.3 (5.3–11.9)	13.5 (0.3)		13.7 (8.3–18-7)***
Zinc	mg	10	21.9 (13.1–33.1)	0 (0.0–4.9)	22.3 (15.5–30.4)	0.8 (0.0–4.2)	73.1** (64.6–80.5)	10.8** (6.0–17.4)	8.7 (0.2)	8.4 (5.5–12.3)	8.6 (0.2)	8.1 (5.6–11.8)	11.7 (0.2)		11.7 (8.2–16.1)***
Iodine	µg	250	20.5 (12.0–31.6)	5.5 (1.5–13.4)	23.1 (16.1–31.3)	10.8 (6.0–17.4)	32.3 (24.4–41.1)	13.8 (8.4–21.0)	201 (10)	186 (92–392)	208 (9)	177 (103–434)	253 (9)		226 (136–493)***
Selenium	µg	90	0 (0.0–4.9)	0 (0.0–4.9)	0 (0.0–2.8)	0 (0.0–2.8)	0 (0.0–2.8)	0 (0.0–2.8)	36.5 (1.1)	35.5 (22.4–53.7)	35.7 (0.9)	34.8 (21.9–54.5)	49.0 (1.0)		47.0 (32.6–70.6)***

*P < 0.05; **P < 0.01, ***P < 0.001 (user vs non-user)

^a^In 2012 the UL for vitamin D was increased from 25 µg/day to 50 µg/day

^b^The UL applies to supplements only

^c^UL for α-tocopherol equivalents (α-TEs). 1 α-TE = 1 mg RRR α-tocopherol

^d^temporary guidance level (TGL) suggested by a Danish expert group [26]

^e^Guidance level (GL) established by a British expert group (Expert group on Vitamins and Minerals 2003) and extrapolated to children by Rasmussen et al. [26]

^f^Niacin equivalents (NEs). 1 NE = 1 mg niacin = 60 mg tryptophan

**Table 7:** Percentages of users and non-users of specific micronutrients among children aged 7–10 years (n = 296) in exceedance of the upper intake level (UL) through the diet alone and when also considering supplement use (% (95% CI) and the mean (SD) and median (95% CI) intakes of micronutrients

Micronutrient		UL	Non-users		Users				Non-users		Users			
			Dietary intake		Dietary intake		Total intake		Dietary intake		Dietary intake		Total intake	
			> UL	> 150%	> UL	> 150%	> UL	> 150%	Mean (SD)	Median (95% CI)	Mean (SD)	Median (95% CI)	Mean (SD)	Median (95% CI)
Retinol	µg	1500	11.5 (6.4–18.5)	3.3 (0.9–8.2)	14.9 (10.0–21.1)	4.6 (2.2–8.9)	20.7 (14.9–27.5)	6.9 (3.6–11.7)	798 (51)	600 (237–1889)	862 (51)	632 (232–2259)	1100 (53)	886 (375–2483)***
Vitamin D	µg	25	0 (0.0–3.1)	0 (0.0–3.1)	0 (0–2.1)	0 (0–2.1)	1.1 (0.1–4.0)	0.5 (0.0–3.1)	3.0 (0.2)	2.2 (1.0- 8.7)	2.6 (0.2)	2.0 (1.0–5.6)	9.3 (0.5)	8.7 (2.5–20.5)***
Vitamin E^b^	α-TE^c^	160	–	–	–	–	0 (0–2.1)	0 (0–2.1)	7.7 (0.2)	7.2 (4.3–12.4)	7.8 (0.2)	7.2 (4.3–13.7)	12.1 (0.3)	11.3 (6.5–19.6)***
Thiamin	mg TGL^d^	25	0 (0.0–3.1)	0 (0.0–3.1)	0 (0–2.1)	0 (0–2.1)	0 (0–2.1)	0 (0–2.1)	1.3 (0.0)	1.2 (0.7–2.0)	1.2 (0.0)	1.2 (0.7–1.8)	1.7 (0.0)	1.8 (1.0–2.4)***
Riboflavin	mg GL^e^	22	0 (0.0–3.1)	0 (0.0–3.1)	0 (0–2.1)	0 (0–2.1)	0 (0–2.1)	0 (0–2.1)	1.7 (0.1)	1.6 (0.8–3.0)	1.7 (0.0)	1.6 (0.9–2.5)	2.2 (0.0)	2.2 (1.2–3.2)***
Niacin	NE^f^	350	0 (0.0–3.1)	0 (0.0–3.1)	0 (0–2.1)	0 (0–2.1)	0 (0–2.1)	0 (0–2.1)	24.8 (0.6)	23.7 (12.8–36.6)	23.3 (0.5)	22.4 (14.5–34.7)	28.7 (0.5)	28.4(19.2–40.2)***
Vitamin B6	mg	10	0 (0.0–3.1)	0 (0.0–3.1)	0 (0–2.1)	0 (0–2.1)	0 (0–2.1)	0 (0–2.1)	1.3 (0.0)	1.3 (0.7–1.9)	1.3 (0.0)	1.2 (0.8–1.9)	1.9 (0.3)	1.9 (1.1–2.7)***
Folic acid^b^	mg	400	–	–	–	–	0 (0–2.1)	0 (0–2.1)	297 (9)	286 (166–458)	288 (7)	270 (169–460)	344 (7)	323 (207–522)***
Vitamin B12	µg GL^e^	1000	0 (0.0–3.1)	0 (0.0–3.1)	0 (0–2.1)	0 (0–2.1)	0 (0–2.1)	0 (0–2.1)	5.7 (0.2)	5.1 (2.1–11.0)	5.6 (0.2)	5.1 (2.3–10.0)	6.4 (0.2)	6.1 (2.9–10.6)**
Vitamin C	mg TGL^d^	500	0 (0.0–3.1)	0 (0.0–3.1)	0 (0–2.1)	0 (0–2.1)	0.6 (0–3.2)	0 (0–2.1)	112 (5)	99 (45–231)	103 (4)	88 (45–195)	137 (6)	122 (61–249)***
Calcium	mg	2500	0 (0.0–3.1)	0 (0.0–3.1)	0 (0–2.1)	0 (0–2.1)	0 (0–2.1)	0 (0–2.1)	1090 (35)	1029 (576–1813)	1072 (24)	1030 (613–1641)	1136 (25)	1097 (657–1731)
Magnesium ^b^	mg	250	–	–	–	–	0 (0–2.1)	0 (0–2.1)	310 (7)	301 (192–434)	304 (6)	287 (191–458)	318 (6)	301 (210–481)
Iron	mg TGL^d^	20	0 (0.0–3.1)	0 (0.0–3.1)	0 (0–2.1)	0 (0–2.1)	6.4** (3.3–11.2)	0.6 (0–3.2)	9.7 (0.2)	9.5 (6.3–13.6)	9.5 (0.2)	9.0 (6.2–14.6)	14.0 (0.3)	13.9 (8.7–20.6)***
Zinc	mg	13	15.2 (9.4–22.7)	0.8 (0–4.4)	14.0 (9.2–20.2)	0 (0–2.1)	45.6** (38.0–53.4)	0.6 (0–3.2)	10.4 (0.3)	9.6 (6.0–15-2)	10.1 (0.2)	9.8 (6.1–14.5)	12.7 (0.2)	12.8 (8.1–17.5)***
Iodine	µg	300	26.4 (18.9–35.0)	7.2 (3.3–13-2)	15.2* (10.2–21.5)	5.3 (2.4–9.8)	26.3 (19.9–33.6)	5.8 (2.8–10.5)	252 (12)	214 (99–535)	223 (7)	213 (111–462)	261 (8)	247 (128–493)*
Selenium	µg	130	0 (0.0–3.1)	0 (0.0–3.1)	0 (0–2.1)	0 (0–2.1)	0 (0–2.1)	0 (0–2.1)	43.7(1.2)	41.0 (23.7–68.0)	41.5 (0.9)	40.5 (24.9–61.3)	53.2 (1.1)	52.0 (31.9–76.2)***

*P < 0.05; **P < 0.01, ***P < 0.001 (user vs non-user)

^a^In 2012 the UL for vitamin D was increased from 25 µg/day to 50 µg/day

^b^The UL applies to supplements only

^c^UL for α-tocopherol equivalents (α-TEs). 1 α-TE = 1 mg RRR α-tocopherol

^d^temporary guidance level (TGL) suggested by a Danish expert group [26]

^e^Guidance level (GL) established by a British expert group (Expert group on Vitamins and Minerals 2003) and extrapolated to children by Rasmussen et al. [26]

^f^Niacin equivalents (NEs). 1 NE = 1 mg niacin = 60 mg tryptophan

In 7–10-year-olds, 12–15% of supplement users and non-users had dietary intakes in exceedance of the ULs for retinol and zinc. However, the proportion of non-users exceeding the UL for iodine was significantly higher (26.4%, *P* = 0.025) than the proportion of users exceeding the UL for iodine through diet alone (15.2%). As for total iodine intake in users, the proportion exceeding UL was similar to that in non-users. Supplement use meant that a significantly higher proportion of users (6.4%, *P* = 0.003, Fisher’s exact test) exceeded the UL for iron compared with non-users (0%).

### Misreporting of energy

In the study population (n = 484), 96.5% were classified as plausible reporters of energy, 2.5% as under-reporters and 1.0% as over-reporters. There was no significant difference in the proportion of misreporters of energy between users and non-users of dietary supplements (*P* = 0.589).

## Discussion

In this study, the use of dietary supplements and the intake of 17 selected micronutrients were investigated in Danish children aged 4–10 years. The aim was to assess whether dietary supplement use leads to micronutrient intakes that exceed the respective ULs. Nearly two-thirds of children in the study had used a dietary supplement in the past year. This is similar to the proportion of Danish adults using dietary supplements (60%) [3, 15], but is a higher proportion compared to children in other European countries [1, 6–8, 31] and in many other developed countries (1.1–45.5%) [32–38]. However, differences in data collection methodology (e.g. duration of reference periods and types of dietary supplements recorded) makes it difficult to compare use of dietary supplements between countries. The reference period of 12 months in the present study and use of any type of dietary supplement (including herbal supplements, fish oil, yeast extracts, probiotics, etc.) may explain the relatively high percentage of supplement users in the present study.

MVMSs were the most popular type of dietary supplement in Danish children, similar to statistics in other Western countries [8, 11, 31, 32, 37]. The widespread use of dietary supplements in Danish children led to a considerable proportion of supplement users exceeding the ULs for retinol, zinc, iodine and TGL for iron. The Danish diet is rich in vitamin A, zinc, and iodine, and for a considerable proportion of the participants (11.5–30.1%), the diet alone provided retinol, zinc, iodine at levels that exceeded the ULs for these three micronutrients. The high iodine intake is mainly due to a high intake of milk which provides nearly half of the iodine in the diet of children. Furthermore, cereals and table salt are other significant sources of iodine (each about 15%). Main contributors of zinc are cereals, meat, and milk providing 73% of the total intake. The high retinol intake is mainly due to liver pâté and liver. About two thirds of vitamin A in the Danish diet is in the form of retinol. Adding a dietary supplement on top of a nutrient-adequate diet, led to an even greater proportion of the study population exceeding the ULs for these three micronutrients, although the proportions of users and non-users exceeding the UL were only significantly different in the case of zinc. However, in the case of retinol, the proportion exceeding the UL by 150% was significantly higher among users than non-users. Also, supplement use led to a significantly larger proportion of users exceeding the UL for iron compared with non-users.

In contrast to surveys of adults where former smokers were more likely to use dietary supplements [15, 37, 39–41], we found that supplement use was significantly higher in children whose parents were never-smokers, whereas the proportion of users among children of former smokers was not significantly different from that of children of smokers. This was confirmed in a logistic regression analysis where we found smoking status to be the only significant variable associated with dietary supplement use, with smokers being less likely to use dietary supplements than never-smokers. Similar associations between parental smoking status and supplement use in children have been reported in NHANES [42] and in a German survey [8]. In other surveys, use of dietary supplements in children were associated with a more prudent diet, healthier lifestyle, higher parental income and education, but also with chronic illness [8, 11, 32, 41, 43]. In adults, dietary supplement use has been linked with other healthy lifestyle choices [41, 44]. In studies on Danish adults other factors were also associated with dietary supplement use, including age, sex, self-perceived health status [15], intention to eat a healthy diet [3], health index and educational attainment [43]. However, in contrast to other studies [41, 45], we did not find significant differences in micronutrient intakes from the diet between users and non-users, nor did the diet quality score differ. The small sample size in the present study could be a reason why we did not find more factors associated with dietary supplement use or any significant differences in dietary intakes. However, this may also be due to differences in the factors associated with dietary supplement use between adults and children and in between countries. Studies show that in younger children, dietary supplement use often reflects the parents’ dietary supplement use [41, 46]. This could also explain why there was no difference in dietary supplement use between boys and girls in this survey, whereas surveys of adults showed that women are more likely to consume dietary supplements [2, 3, 15]. We observed a non-significant higher proportion of children using MVMSs in 4–6 year olds compared to 7–10 year olds which is in accordance with findings in other surveys [11, 33].

Compared to other European countries, Danish children have a high dietary iodine intake [1]. This is despite a relatively low iodine fortification level (13 µg iodine/kg salt until mid-2019) in Denmark and fortification of only table salt and salt added to bread and bakery products [47, 48]. High intakes of dairy products, bread, cereals and, to a lesser extent, marine foods may explain the high iodine content of the diet of Danish children [17, 48]. Tap water is also an important source of iodine in Denmark, although the iodine content of tap water varies with geography [47]. In the present survey, a considerable proportion of children had a dietary iodine intake that exceeded the UL. When adding the iodine from supplements on top of a high dietary intake, about a third of 4–6-year-olds and 26% of 7–10-year-olds exceeded the UL for iodine. Following reporting of the DANSDA survey results [17], the Danish Veterinary and Food Administration recommended removing iodine from MVMSs marketed for children. Several manufacturers have subsequently revised the formulation of their MVMSs products for children. The Danish Health Authority recommends parents avoid giving MVMSs containing iodine to 3–10-year-old children who drink milk [12]. However, the mandatory iodine fortification programme was revised in 2019 and the iodine content of salt changed from 13 to 20 mg/kg. Thus, many children may still be at risk of exceeding the UL for iodine, although use of MVMSs may contribute less to excess iodine intake now.

It has previously been reported that Danish children have a high zinc intake (95% percentile > UL) compared with children in other European countries [1]. In the present survey, 22% of the children aged 4–6 years and 14–15% aged 7–10 years had a dietary zinc intake that exceeded the UL for the respective age groups. With even more zinc provided by dietary supplements, 73% of 4–6-year-old users and 45% of 7–10-year-old users exceeded the UL. Similar findings have been reported children in other high-income countries [33, 49, 50]. However, exceedance of the UL for zinc does not appear to be associated with adverse effects and is therefore not considered an issue [24, 51].

Iron intake exceeded TGL among supplement users in both age groups. The higher prevalence of excess iron intake in 4–6-year-olds may be due to provision of the same vitamin/mineral supplements to 4–6 and 7–10 years-olds. However, a significant proportion of children in the present study concurrently exceeded the TGL due to the small margin between the RI and TGL. Chronic excess iron intake could be problematic as there is no biological mechanism for excreting excess iron [52] and the consequence can be iron overload. Iron overload can lead to health issues such as gastrointestinal bleeding, diarrhoea and nausea [26]. As no UL for iron has been established for children in Europe, we chose to use the TGL suggested by Rasmussen et al. [26]. We are aware that the US Institute of Medicine (IoM) has published a UL for iron of 40 mg/day for children. No children in this study exceeded the IoM UL for iron. However, as discussed by Rasmussen et al. [26] the IoM UL is based on observations of acute adverse gastrointestinal effects and the UL does not consider population groups vulnerable to iron overload. Rasmussen et al. extrapolated the provisional maximum tolerable daily intake (PMTDI) established at 0.8 mg/kg bodyweight by FAO/WHO Joint Expert Group on Food Additives in 2003 to children based on surface area [26].

We also found that a high proportion (12–30%) of children exceeded the UL for retinol. When looking at the diet alone this was especially true among 4–6-year-olds. Among supplement users, 43.1% of 4–6-year-olds exceeded the UL for retinol, whereas 20.7% of 7–10-year-olds exceeded the UL. The diet of Danish children is generally rich in vitamin A due to frequent consumption of food items such a pork liver pâté, liver, and carrots. Especially the youngest children have a vitamin A-rich diet [17]. Limiting further retinol intake from dietary supplements would help prevent toxic effects of prolonged excessive vitamin A intake. Long term hypervitaminosis A can lead to hepatotoxicity, hypercalcemia, and skin and bone changes [53]; however, data on the long-term effects of excessive retinol intake in children is scarce at present [33]. To our knowledge, no adverse effects from excessive retinol intake in Danish children have been reported. However, adverse events related to dietary supplement use are not systematically recorded in Denmark. The 5^th^ percentile for vitamin A intake in this study population (4–10 years old children) is approx. 400 RE, which is the RI for 6–9-year-olds, and the median intake is 2.5-fold the RI. Thus, reducing the amount of vitamin A in dietary supplements is unlikely to lead to widespread vitamin A insufficiency in Danish children.

Children whose diet provided vitamin and/or mineral intakes above ULs had a higher energy intake and consumed more foods rich in iodine (dairy products, bread, cereals, salt), retinol (pork liver pâté, liver) and zinc (meat, bread, cereals, dairy products) than children whose diets did not provide excessive levels of micronutrients (data not shown). Excess micronutrient intakes obtained through the diet is less concerning due to variation in diet over time.

Children are particularly vulnerable to excess intake of micronutrients because they are growing and because of the small margin between optimal intake and excess intake [35]. The ULs for children are extrapolated from adult values on the basis of metabolic body weight [24, 54] and the margins between the Estimated Average Requirements and ULs are smaller for young children than for adults due to the use of a greater uncertainty factor in the calculation of ULs for children [54]. This could partly explain why a larger proportion of children than adults exceed ULs through the diet and why a relatively larger proportion of dietary supplement users amongst children exceed ULs [1]. Another factor contributing to excess intakes is consumption of fortified foods. As fortified foods become more common in Denmark, these foods also need to be regarded as significant sources of micronutrients.

The strength of the Danish National Survey of Diet and Physical Activity is that it is based on a random sample of the general population, it has a high response rate among children, the reference period for dietary supplement use was 12 months, and the habitual diet was recorded for 7 days. Whilst a reference period of 12 months may be a strength in that it accounts for supplements consumed episodically, it may also be susceptible to difficulties in recalling supplements used occasionally or many months ago. It may also be considered a limitation that any supplement use in the past 12 months categorizes a person as a user. This may partly explain the high proportion of users in Denmark compared to other countries. Measuring dietary intake in children is difficult and often prone to reporting error with under-reporting of energy being frequent, but some over-reporting also occurs [55, 56]. However, in this study, 96.5% were classified as plausible reporters of energy making misreporting a minor issue. The contents of micronutrients such as iodine and selenium are highly variable in some foods. However, the national food composition database in Denmark is updated regularly, the vitamin and mineral contents of many foods have been analysed and seasonal variation is taken into account when collecting samples for analyses. Missing data in databases can lead to underestimation of the intake of micronutrients, however, there are few missing values in the food composition data used in DANSDA.

Another limitation is the calculation using a generic MVMS instead of the specific MVMS taken by participants. Two studies found greater accuracy with using more specific types of MVMSs in calculations of micronutrient intakes from supplements compared to using a generic MVMS [57, 58]. Furthermore, due to the small sample size, we did not distinguish between daily, frequent and occasional users of dietary supplements, nor did we exclude mis-reporters of dietary intake from the analyses. Improvements in methodology will make the estimation of micronutrient intakes from dietary supplements more accurate in future surveys compared to the present survey.

In conclusion, MVMSs are the most commonly consumed dietary supplement. Most dietary supplements users consumed only one dietary supplement. Only smoking status of the parent was significantly associated with supplement use, with children of non-smoking parents more likely to be supplement users. There were no differences in dietary intakes of micronutrients between dietary supplement users and non-users. However, a considerable proportion of children consumed excess amounts of retinol, iodine, and zinc through their diet. With dietary supplements further increasing intake of retinol, iodine, and zinc, some children reached very high intakes of these three micronutrients. The consequences of chronic excessive intake of retinol, iodine and zinc in children is unknown. Excess zinc and iodine may be less of a problem than excess iron and retinol, which can cause longer-term health issues in the form of hepatic fibrosis [53, 59]. Supplement use also led to excess iron intake in some children, particular in the youngest children. We acknowledge that the diet of some children may provide insufficient amounts of zinc, iodine, and iron (data not shown); thus, for those consuming a suboptimal diet, a dietary supplement may be beneficial. However, median intakes of zinc and iodine were above the RIs, indicating that the prevalence of inadequate zinc and iodine intakes is low. For most Danish 4–10-year-olds, the diet seems to provide enough micronutrients with the exception of vitamin D. Thus, most Danish children do not need dietary supplements.

## Data Availability

In accordance with Danish law and GDPR, the data and the analytical scripts used in the present study can only be accessed through the servers at The Technical University of Denmark and requires a Disclosure Declaration. Access and a Disclosure Declaration can be granted upon request if the applicant fulfils the criteria for access. The Technical University of Denmark can be contacted by email: camp@food.dtu.dk or apbj@food.dtu.dk.
